# Toscana Virus Isolated from Sandflies, Tunisia

**DOI:** 10.3201/eid1902.121463

**Published:** 2013-02

**Authors:** Laurence Bichaud, Khalil Dachraoui, Géraldine Piorkowski, Ifhem Chelbi, Gregory Moureau, Saifedine Cherni, Xavier De Lamballerie, Sonia Sakhria, Rémi N. Charrel, Elyes Zhioua

**Affiliations:** Author affiliations: Aix-Marseille University (AMU, IRD, EHESP), Marseille, France (L. Bichaud, G. Piorkowski, G. Moureau, X. De Lamballerie, R.N. Charrel);; IHU Méditerranée Infection, Marseille (L. Bichaud, X. De Lamballerie, R.N. Charrel);; Institut Pasteur, Tunis, Tunisia (K. Dachraoui, I. Chelbi, S. Cherni, S. Sakhria, E. Zhioua)

**Keywords:** Toscana virus, sandflies, viruses, Tunisia, Phlebotomus perniciosus, Phlebotomus perfiliewi, Phlebovirus, Bunyaviridae

**To the Editor:** Toscana virus (TOSV; genus *Phlebovirus*, family *Bunyaviridae*) is transmitted by sandflies, mostly the species *Phlebotomus perniciosus* and *P. perfiliewi* (*1*). Initially discovered in central Italy, TOSV was recently identified in other European countries (i.e., Portugal, Spain, France, Croatia, and Turkey) (*2*). TOSV is a primary cause of aseptic meningitis during warm months (*2*). A seroprevalence study suggested that TOSV is present in Tunisia and may cause neuroinvasive infections, but definitive evidence of TOSV circulation has not been possible because it is difficult to distinguish from the antigenically related phlebovirus Punique virus (*3*,*4*); both viruses are members of the species *Sandfly fever Naples virus*. We investigated the prevalence of TOSV among sandflies in northern Tunisia.

A total of 5,288 sandflies (3,547 females, 1,740 males) were collected during June–October 2010 by using CDC light traps (John W. Hock Company, Gainesville, FL, USA) at Utique (37°08′N, 7°74′E), a focus for visceral leishmaniasis in northern Tunisia. Sandflies were separated by sex and trapping nights and pooled with >30 specimens by pool. Pools were processed as described (*4*) and subjected to PCR detection of phlebovirus RNA targeting 2 genes independently (*4*–*6*) and virus isolation onto Vero cells. Of 249 pools processed, 8 strains of phleboviruses were isolated: 2 TOSV, 3 Punique virus, and 3 other phleboviruses currently being characterized. 

TOSV strains were obtained from 2 pools of sandflies trapped in September 2010, T152 and T166, consisting of 30 males and 30 females, respectively. These pools were positive for TOSV RNA by sequencing of 2 PCR products (201-nt and 280-nt sections in the large [L] and small [S] gene segments, respectively). Supernatant of the third passage was prepared for electron microscopy, which showed spherical and pleomorphic structures, 80–120 nm in diameter, compatible with viruses of the family *Bunyaviridae*. Complete genome sequencing was then done by using the Ion PGM Sequencer (Life Technologies SAS, Saint Aubin, France) (*7*); a total of 165,307 reads were obtained, of which 135,700 matched with the sequence of TOSV Iss.PhL3 used as reference. The viral genome of TOSV Tunisia-2010-T152 (GenBank accession nos. JX867534–JX867536) was composed of 12,488 nt; the complete sequence consisted of 1,869 nt, 4,215 nt, and 6,404 nt for the S, medium [M], and L RNA segments, respectively. The partial S sequence of the TOSV Tunisia-2010-T166 strain (GenBank accession nos. JX867537–JX867539) was identical to that of T152, but 1 synonymous mutation was observed in the partial L sequence and 1 nonsynonymous mutation in the partial M sequence (I906V).

The TOSV Tunisia-2010-T152 strain was aligned with homologous sequences retrieved from the GenBank database. Genetic distances were calculated at the amino acid and nucleotide levels by using the p-distance algorithm (Technical Appendix Tables 1–3). Phylogenetic studies were performed by using the neighbor-joining method in MEGA5 (*8*) (Figure). The robustness of the nodes was tested by 1,000 bootstrap replications. We found that TOSV Tunisia-2010-T152 was most closely related to the prototype strain from Italy, Iss.PhL3, with nucleotide/amino acid distances of 0.031/0.052, 0.032/0.073, and 0.039/0.012 for the S, M, and L RNA sequences, respectively. Together, these genetic distances and phylogram topologies indicate that TOSV Tunisia-2010-T152 is most closely related to strains within the Italian lineage, although it may represent a distinct sublineage, more distantly related to strains belonging to the Spanish lineage (*9*).

**Figure:**
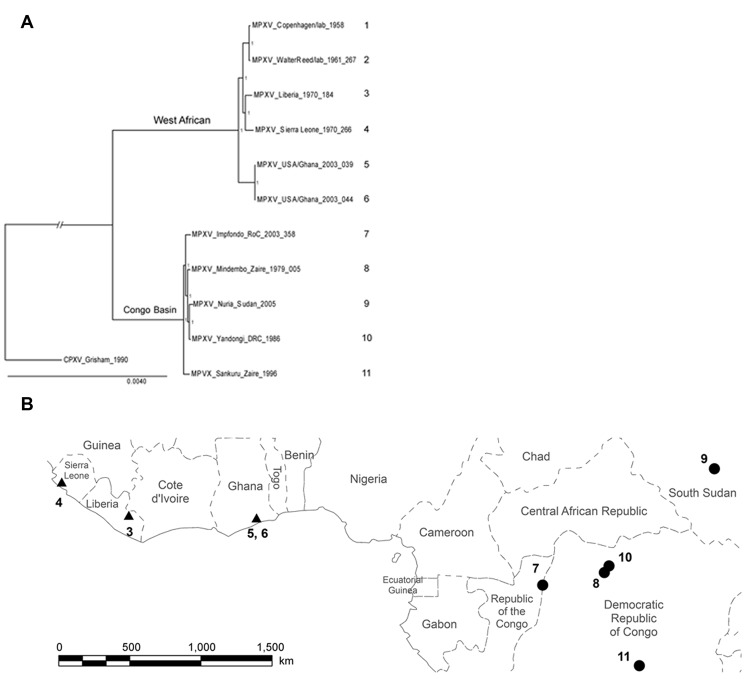
Phylogenetic analysis of 3 segments of Toscana virus (TOSV) isolates from pools of sandflies collected in Tunisia and homologous sequences of other selected phleboviruses. A) Large segments; B) medium segments; C) small segments. Sequences are identified by virus name or acronym, strain name, and GenBank accession number. Scale bars indicate nucleotide substitutions per site. TEHV, Tehran virus; SFNV, sandfly fever Naples virus; PNQV, Punique virus; RVFV, Rift Valley fever virus; ARBV, Arbia virus; CHIOS, phlebovirus Chios-A; CFUV, Corfou virus; SFSV, sandfly fever Sicilian virus; UUKV, Uukuniemi virus.

Concomitantly with virus isolation, the phenology of sandfly species was studied during May–November 2010. Sandflies were identified, and the density was calculated as described (*10*). Most of the sandflies belonged to the subgenus *Larroussius* (98.3%). *P. perniciosus* sandflies were the most abundant species (71.74%), followed by *P. longicuspis* (17.47%) and *P. perfiliewi* (8.82%). Other sandfly species, such as *Phlebotomus* (*Phlebotomus*) *papatasi*, *Phlebotomus* (*Paraphlebotomus*) *sergenti*, *Sergentomyia minuta parotti*, *S. christophersi*, and *S. antennata* were found, but these were much less abundant. The phenology of 3 main sandfly species showed 2 main peaks: 1 small peak in June and a second, larger peak during September–October (Technical Appendix Figure).

In summary, of a total of 5,288 sandflies collected, 2 pools were positive for TOSV, yielding an infection rate of 0.03%. A similar infection rate was observed in Spain (0.05%) (*9*); however, the infection rates in Italy (0.22%) and in France (0.29%) are substantially higher (*1*,*6*). The isolation of TOSV from male and female sandflies suggests transovarial transmission in nature, as reported in Italy and Spain (*1*,*9*). In southern European countries, TOSV is mostly transmitted by *P*. *perniciosus* and *P. perfiliewi* sandflies (*1**,**6**,**9*), whereas *P. perniciosus*, *P. longicuspis*, and *P. perfiliewi* are the most abundant sandfly species in northern Tunisia. It is therefore probable that TOSV is transmitted by sandfly species of the subgenus *Larroussius*.

We found that 2 phleboviruses belonging to the *Sandfly fever Naples virus* species, TOSV and Punique virus, are cocirculating in northern Tunisia. This finding calls for further investigation of these viruses’ potential effect on human health in this area.

Technical Appendix Genetic pairwise distances between Toscana virus strains and selected phleboviruses and phenology of sandflies collected in Utique, Governorate of Bizerte, Tunisia.
