# Supplementary material for: Toscana Virus Isolated from Sandflies, Tunisia
Source: Emerg Infect Dis. 2013 Feb;19(2):322–4. doi: 10.3201/eid1902.121463 (PMC3559066; doi:10.3201/eid1902.121463)
Supplement: Technical Appendix — Genetic pairwise distances between Toscana virus strains and selected phleboviruses and phenology of sandflies collected in Utique, Governorate of Bizerte, Tunisia. [file 12-1463-Techapp_s1.pdf]

Article DOI: http://dx.doi.org/10.3201/eid1902.121463

# Toscana Virus Isolated from Sandflies, Tunisia

## Technical Appendix

Technical Appendix Table 1. Genetic pairwise distances between Tunisian Toscana virus (TOSV) strains and selected phleboviruses in large RNA segment.

| N | Virus | 1 | 2 | 3 | 4 | 5 | 6 | 7 | 8 | 9 | 10 | 11 | 12 | 13 | 14 | 15 | 16 | 17 | 18 | 19 | 20 | 21 | 22 | 23 | 24 | 25 | 26 | 27 | 28 | 29 |
|---|---|---|---|---|---|---|---|---|---|---|---|---|---|---|---|---|---|---|---|---|---|---|---|---|---|---|---|---|---|---|
| | | | | | | | | | | | | | | | | | | | | | | | | | | | | | | *Nucleotide sequences* |
| 1 | TOSV_Tunisia2010_T152_Complete | | 0.002 | 0.039 | 0.067 | 0.146 | 0.146 | 0.179 | 0.147 | 0.269 | 0.328 | 0.299 | 0.333 | 0.338 | 0.266 | 0.289 | 0.271 | 0.284 | 0.284 | 0.423 | 0.413 | 0.369 | 0.418 | 0.383 | 0.399 | 0.356 | 0.383 | 0.380 | 0.482 | 0.575 |
| 2 | TOSV_Tunisie2010_T166_Partial | 0.000 | | 0.059 | 0.059 | 0.166 | 0.168 | 0.179 | 0.156 | 0.301 | 0.328 | 0.301 | 0.333 | 0.338 | 0.275 | 0.289 | 0.271 | 0.284 | 0.284 | 0.423 | 0.413 | 0.390 | 0.418 | 0.390 | 0.399 | 0.358 | 0.384 | 0.368 | 0.501 | 0.588 |
| 3 | TOSV_ISS.Phl.3_NC_006319 | 0.012 | 0.006 | | 0.017 | 0.149 | 0.149 | 0.174 | 0.150 | 0.272 | 0.333 | 0.293 | 0.338 | 0.333 | 0.273 | 0.308 | 0.259 | 0.308 | 0.308 | 0.413 | 0.403 | 0.373 | 0.413 | 0.379 | 0.394 | 0.356 | 0.385 | 0.382 | 0.461 | 0.573 |
| 4 | TOSV_France_DQ1500590 | 0.011 | 0.006 | 0.006 | | 0.153 | 0.155 | 0.174 | 0.150 | 0.290 | 0.333 | 0.293 | 0.338 | 0.333 | 0.265 | 0.308 | 0.259 | 0.308 | 0.308 | 0.413 | 0.403 | 0.366 | 0.413 | 0.385 | 0.394 | 0.356 | 0.385 | 0.348 | 0.473 | 0.583 |
| 5 | TOSV_AR_France_DQ65607 | 0.032 | 0.036 | 0.035 | 0.034 | | 0.008 | 0.020 | 0.027 | 0.265 | 0.353 | 0.329 | 0.318 | 0.338 | 0.284 | 0.313 | 0.246 | 0.348 | 0.348 | 0.433 | 0.423 | 0.389 | 0.393 | 0.373 | 0.399 | 0.384 | 0.401 | 0.365 | 0.464 | 0.579 |
| 6 | TOSV_France_H/IMTSSA_FJ153281 | 0.031 | 0.036 | 0.034 | 0.034 | 0.002 | | 0.015 | 0.025 | 0.266 | 0.348 | 0.327 | 0.318 | 0.338 | 0.280 | 0.308 | 0.251 | 0.338 | 0.338 | 0.433 | 0.423 | 0.389 | 0.393 | 0.374 | 0.394 | 0.382 | 0.401 | 0.361 | 0.466 | 0.580 |
| 7 | TOSV_ESPh-GR79_GU183147 | 0.030 | 0.030 | 0.015 | 0.015 | 0.000 | 0.000 | | 0.020 | 0.353 | 0.353 | 0.353 | 0.318 | 0.328 | 0.333 | 0.303 | 0.259 | 0.338 | 0.333 | 0.438 | 0.428 | 0.428 | 0.398 | 0.433 | 0.408 | 0.408 | 0.478 | 0.438 | 0.542 | 0.577 |
| 8 | TOSV_Spain_EsPhGR40_FJ153280 | 0.031 | 0.036 | 0.034 | 0.034 | 0.003 | 0.003 | 0.000 | | 0.268 | 0.343 | 0.337 | 0.318 | 0.338 | 0.286 | 0.294 | 0.244 | 0.333 | 0.333 | 0.443 | 0.433 | 0.393 | 0.403 | 0.378 | 0.390 | 0.378 | 0.399 | 0.368 | 0.471 | 0.573 |
| 9 | Massilia_W_EU725771 | 0.178 | 0.196 | 0.181 | 0.198 | 0.176 | 0.176 | 0.269 | 0.175 | | 0.030 | 0.042 | 0.358 | 0.333 | 0.293 | 0.368 | 0.279 | 0.343 | 0.348 | 0.413 | 0.403 | 0.360 | 0.398 | 0.332 | 0.394 | 0.349 | 0.375 | 0.417 | 0.487 | 0.580 |
| 10 | Massilia_BP_DQ656073 | 0.254 | 0.254 | 0.269 | 0.269 | 0.284 | 0.284 | 0.284 | 0.284 | 0.015 | | 0.000 | 0.338 | 0.318 | 0.343 | 0.368 | 0.313 | 0.338 | 0.343 | 0.403 | 0.393 | 0.393 | 0.398 | 0.328 | 0.413 | 0.413 | 0.443 | 0.468 | 0.552 | 0.577 |
| 11 | Massilia_BM_DQ656072 | 0.202 | 0.202 | 0.208 | 0.208 | 0.220 | 0.220 | 0.284 | 0.220 | 0.006 | 0.000 | | 0.338 | 0.318 | 0.293 | 0.368 | 0.277 | 0.338 | 0.343 | 0.403 | 0.393 | 0.366 | 0.398 | 0.347 | 0.408 | 0.362 | 0.380 | 0.398 | 0.515 | 0.594 |
| 12 | PNQV_P6B2_2008_GQ165519 | 0.269 | 0.269 | 0.254 | 0.254 | 0.254 | 0.254 | 0.254 | 0.254 | 0.254 | 0.239 | 0.239 | | 0.055 | 0.398 | 0.323 | 0.333 | 0.398 | 0.398 | 0.468 | 0.458 | 0.458 | 0.468 | 0.428 | 0.453 | 0.453 | 0.458 | 0.458 | 0.542 | 0.562 |
| 13 | PNQV_P1B4_2008_FJ848989 | 0.239 | 0.239 | 0.224 | 0.224 | 0.224 | 0.224 | 0.224 | 0.224 | 0.224 | 0.239 | 0.239 | 0.045 | | 0.388 | 0.318 | 0.318 | 0.388 | 0.388 | 0.473 | 0.463 | 0.463 | 0.473 | 0.408 | 0.443 | 0.443 | 0.453 | 0.458 | 0.552 | 0.547 |
| 14 | SFNV_Poona_EF095548 | 0.180 | 0.185 | 0.186 | 0.181 | 0.186 | 0.186 | 0.254 | 0.186 | 0.230 | 0.358 | 0.232 | 0.328 | 0.328 | | 0.249 | 0.267 | 0.010 | 0.000 | 0.428 | 0.418 | 0.349 | 0.433 | 0.360 | 0.418 | 0.362 | 0.409 | 0.354 | 0.501 | 0.586 |
| 15 | SFNV_30451_GQ165528 | 0.224 | 0.224 | 0.209 | 0.209 | 0.194 | 0.194 | 0.194 | 0.194 | 0.358 | 0.343 | 0.343 | 0.284 | 0.284 | 0.090 | | 0.249 | 0.249 | 0.249 | 0.493 | 0.483 | 0.483 | 0.463 | 0.433 | 0.428 | 0.428 | 0.433 | 0.473 | 0.527 | 0.552 |
| 16 | TEHV_I_47_GQ165522 | 0.143 | 0.143 | 0.143 | 0.143 | 0.149 | 0.149 | 0.119 | 0.149 | 0.190 | 0.299 | 0.196 | 0.299 | 0.269 | 0.155 | 0.164 | | 0.294 | 0.294 | 0.458 | 0.458 | 0.414 | 0.443 | 0.352 | 0.455 | 0.368 | 0.392 | 0.370 | 0.497 | 0.572 |
| 17 | SFNV_Algeria_A5_GU183867 | 0.254 | 0.254 | 0.269 | 0.269 | 0.269 | 0.269 | 0.269 | 0.269 | 0.373 | 0.358 | 0.358 | 0.328 | 0.328 | 0.015 | 0.104 | 0.224 | | 0.010 | 0.438 | 0.428 | 0.428 | 0.443 | 0.428 | 0.433 | 0.433 | 0.468 | 0.478 | 0.557 | 0.602 |
| 18 | SFNV_Algeria_A6_GU183868 | 0.239 | 0.239 | 0.254 | 0.254 | 0.254 | 0.254 | 0.254 | 0.254 | 0.373 | 0.358 | 0.358 | 0.328 | 0.328 | 0.000 | 0.090 | 0.209 | 0.015 | | 0.428 | 0.418 | 0.418 | 0.433 | 0.428 | 0.428 | 0.428 | 0.468 | 0.483 | 0.562 | 0.597 |
| 19 | SFSV_Kabylia_F16_GU183869 | 0.507 | 0.507 | 0.507 | 0.507 | 0.507 | 0.507 | 0.507 | 0.507 | 0.433 | 0.433 | 0.433 | 0.537 | 0.522 | 0.522 | 0.537 | 0.493 | 0.522 | 0.522 | | 0.020 | 0.010 | 0.075 | 0.388 | 0.274 | 0.274 | 0.279 | 0.438 | 0.507 | 0.612 |
| 20 | SFSV_Algeria_Ph_ariasi_EU240882 | 0.478 | 0.478 | 0.463 | 0.463 | 0.463 | 0.463 | 0.463 | 0.463 | 0.418 | 0.418 | 0.418 | 0.507 | 0.493 | 0.507 | 0.493 | 0.463 | 0.507 | 0.507 | 0.060 | | 0.010 | 0.075 | 0.388 | 0.274 | 0.274 | 0.279 | 0.438 | 0.527 | 0.617 |
| 21 | SFSV_Sabin_EF095551 | 0.389 | 0.411 | 0.389 | 0.390 | 0.384 | 0.384 | 0.478 | 0.384 | 0.346 | 0.403 | 0.363 | 0.507 | 0.493 | 0.377 | 0.507 | 0.387 | 0.507 | 0.507 | 0.030 | 0.030 | | 0.065 | 0.357 | 0.263 | 0.261 | 0.285 | 0.357 | 0.477 | 0.614 |
| 22 | Cyprus_virus_AY962268 | 0.478 | 0.478 | 0.478 | 0.478 | 0.478 | 0.478 | 0.478 | 0.478 | 0.403 | 0.403 | 0.403 | 0.507 | 0.493 | 0.507 | 0.507 | 0.463 | 0.507 | 0.507 | 0.045 | 0.045 | 0.015 | | 0.398 | 0.264 | 0.264 | 0.274 | 0.438 | 0.542 | 0.612 |
| 23 | RVFV_Smithburn_DQ375430 | 0.323 | 0.333 | 0.318 | 0.322 | 0.328 | 0.328 | 0.403 | 0.328 | 0.292 | 0.358 | 0.315 | 0.418 | 0.418 | 0.344 | 0.463 | 0.327 | 0.478 | 0.478 | 0.388 | 0.373 | 0.319 | 0.358 | | 0.380 | 0.354 | 0.383 | 0.361 | 0.487 | 0.577 |
| 24 | CHIOS_A_AY293623 | 0.479 | 0.479 | 0.479 | 0.479 | 0.479 | 0.479 | 0.493 | 0.479 | 0.465 | 0.478 | 0.465 | 0.507 | 0.493 | 0.493 | 0.507 | 0.479 | 0.507 | 0.507 | 0.239 | 0.239 | 0.211 | 0.209 | 0.408 | | 0.014 | 0.202 | 0.465 | 0.512 | 0.606 |
| 25 | CFUV_PA_Ar_814_GQ165521 | 0.381 | 0.381 | 0.381 | 0.381 | 0.375 | 0.375 | 0.493 | 0.375 | 0.375 | 0.478 | 0.375 | 0.507 | 0.493 | 0.381 | 0.507 | 0.363 | 0.507 | 0.507 | 0.239 | 0.239 | 0.149 | 0.209 | 0.351 | 0.014 | | 0.230 | 0.384 | 0.503 | 0.612 |
| 26 | Utique_P6B1_2008_GU233649 | 0.399 | 0.399 | 0.399 | 0.399 | 0.399 | 0.399 | 0.507 | 0.399 | 0.381 | 0.478 | 0.381 | 0.522 | 0.507 | 0.399 | 0.522 | 0.393 | 0.522 | 0.522 | 0.239 | 0.239 | 0.185 | 0.194 | 0.333 | 0.056 | 0.089 | | 0.397 | 0.496 | 0.598 |
| 27 | ARBV_Ph_1_35_M6_DQ862467 | 0.364 | 0.327 | 0.364 | 0.316 | 0.364 | 0.364 | 0.448 | 0.364 | 0.379 | 0.507 | 0.351 | 0.522 | 0.507 | 0.328 | 0.493 | 0.315 | 0.522 | 0.522 | 0.552 | 0.522 | 0.384 | 0.522 | 0.355 | 0.563 | 0.423 | 0.440 | | 0.499 | 0.602 |
| 28 | UUKV_NC_005214 | 0.524 | 0.560 | 0.524 | 0.542 | 0.529 | 0.529 | 0.657 | 0.529 | 0.524 | 0.657 | 0.571 | 0.672 | 0.657 | 0.563 | 0.687 | 0.577 | 0.672 | 0.672 | 0.672 | 0.701 | 0.557 | 0.672 | 0.518 | 0.662 | 0.595 | 0.583 | 0.570 | | 0.580 |
| 29 | Gouleako_virus_F23_K1_EF423167 | 0.696 | 0.719 | 0.701 | 0.710 | 0.701 | 0.701 | 0.701 | 0.701 | 0.723 | 0.746 | 0.743 | 0.731 | 0.716 | 0.703 | 0.731 | 0.725 | 0.716 | 0.716 | 0.791 | 0.791 | 0.770 | 0.791 | 0.723 | 0.746 | 0.749 | 0.754 | 0.755 | 0.720 | |
| | | *Amino acid sequences* | | | | | | | | | | | | | | | | | | | | | | | | | | | | |

Technical Appendix Table 2. Genetic pairwise distances between Tunisian Toscana virus (TOSV) strains and selected phleboviruses in medium RNA segment.

| N° | Virus | 1 | 2 | 3 | 4 | 5 | 6 | 7 | 8 | 9 | 10 | 11 | 12 | 13 | 14 | 15 | 16 |
|---|---|---|---|---|---|---|---|---|---|---|---|---|---|---|---|---|---|
| | | | | | | | | | | | | | | | | | *Nucleotide sequences* |
| 1 | TOSV_Tunisia2010_T152_Complete | | 0.002 | 0.032 | 0.176 | 0.173 | 0.178 | 0.437 | 0.449 | 0.397 | 0.397 | 0.401 | 0.527 | 0.587 | 0.526 | 0.632 | 0.680 |
| 2 | TOSV_Tunisie2010_T166_partial | 0.010 | | 0.022 | 0.175 | 0.177 | 0.177 | 0.434 | 0.449 | 0.415 | 0.402 | 0.419 | 0.530 | 0.550 | 0.526 | 0.614 | 0.655 |
| 3 | TOSV_ISSPhl3_NC006320 | 0.073 | 0.040 | | 0.182 | 0.179 | 0.183 | 0.437 | 0.437 | 0.399 | 0.395 | 0.392 | 0.523 | 0.590 | 0.527 | 0.631 | 0.678 |
| 4 | TOSV_France_H/IMTSSA_FJ153284 | 0.423 | 0.374 | 0.427 | | 0.007 | 0.055 | 0.434 | 0.416 | 0.394 | 0.405 | 0.404 | 0.527 | 0.580 | 0.543 | 0.629 | 0.681 |
| 5 | TOSV_AR_France_EF65636 | 0.415 | 0.384 | 0.419 | 0.020 | | 0.057 | 0.436 | 0.418 | 0.393 | 0.410 | 0.404 | 0.528 | 0.582 | 0.543 | 0.629 | 0.680 |
| 6 | TOSV_Spain_EsPhGR40_FJ153283 | 0.432 | 0.404 | 0.428 | 0.150 | 0.154 | | 0.443 | 0.416 | 0.396 | 0.414 | 0.416 | 0.530 | 0.585 | 0.552 | 0.627 | 0.678 |
| 7 | Massilia_W_EU725772 | 0.728 | 0.722 | 0.732 | 0.732 | 0.733 | 0.743 | | 0.243 | 0.431 | 0.397 | 0.413 | 0.559 | 0.583 | 0.566 | 0.625 | 0.677 |
| 8 | PNQV_P1B4_2008_FJ848988 | 0.725 | 0.711 | 0.716 | 0.716 | 0.716 | 0.716 | 0.520 | | 0.421 | 0.413 | 0.429 | 0.529 | 0.578 | 0.531 | 0.601 | 0.652 |
| 9 | SFNV_Poona_HM566177 | 0.700 | 0.705 | 0.707 | 0.690 | 0.680 | 0.682 | 0.726 | 0.700 | | 0.403 | 0.178 | 0.546 | 0.573 | 0.547 | 0.635 | 0.682 |
| 10 | TEHV_I47_AY129732 | 0.619 | 0.646 | 0.619 | 0.657 | 0.667 | 0.657 | 0.670 | 0.696 | 0.713 | | 0.378 | 0.525 | 0.563 | 0.558 | 0.623 | 0.650 |
| 11 | SFNV_Sabin_AY129733 | 0.679 | 0.674 | 0.679 | 0.687 | 0.672 | 0.702 | 0.695 | 0.720 | 0.359 | 0.634 | | 0.550 | 0.537 | 0.526 | 0.653 | 0.636 |
| 12 | SFSV_91045I_AY129740 | 0.776 | 0.785 | 0.765 | 0.745 | 0.755 | 0.765 | 0.806 | 0.816 | 0.760 | 0.755 | 0.750 | | 0.524 | 0.394 | 0.592 | 0.639 |
| 13 | RVFV_NC_014396 | 0.846 | 0.766 | 0.846 | 0.843 | 0.845 | 0.846 | 0.851 | 0.869 | 0.828 | 0.820 | 0.797 | 0.755 | | 0.497 | 0.658 | 0.681 |
| 14 | CFUV_PaAr814_AY129744 | 0.800 | 0.800 | 0.790 | 0.820 | 0.820 | 0.810 | 0.820 | 0.780 | 0.776 | 0.830 | 0.765 | 0.653 | 0.768 | | 0.577 | 0.617 |
| 15 | UUKV_NC_005220 | 0.887 | 0.823 | 0.882 | 0.883 | 0.880 | 0.875 | 0.876 | 0.900 | 0.886 | 0.873 | 0.875 | 0.845 | 0.883 | 0.796 | | 0.692 |
| 16 | Gouleako_virus_A5CI_2004_HQ541 | 0.905 | 0.883 | 0.906 | 0.907 | 0.908 | 0.907 | 0.915 | 0.928 | 0.909 | 0.890 | 0.857 | 0.906 | 0.904 | 0.866 | 0.917 | |
| | | *Amino acid sequences* | | | | | | | | | | | | | | | |

Technical Appendix Table 3. Genetic pairwise distances between Tunisian Toscana virus (TOSV) strains and selected phleboviruses in small RNA segment.

| N° | Virus | 1 | 2 | 3 | 4 | 5 | 6 | 7 | 8 | 9 | 10 | 11 | 12 | 13 | 14 | 15 | 16 | 17 | 18 | 19 | 20 | 21 | 22 | 23 | 24 | 25 | 26 | 27 | 28 |
|---|---|---|---|---|---|---|---|---|---|---|---|---|---|---|---|---|---|---|---|---|---|---|---|---|---|---|---|---|---|
| | | | | | | | | | | | | | | | | | | | | | | | | | | | | | *Nucleotide sequences* |
| 1 | TOSV_Tunisia2010_T152_Complete | | 0.000 | 0.031 | 0.051 | 0.136 | 0.134 | 0.142 | 0.134 | 0.236 | 0.236 | 0.228 | 0.215 | 0.238 | 0.377 | 0.212 | 0.200 | 0.216 | 0.219 | 0.225 | 0.236 | 0.223 | 0.521 | 0.518 | 0.563 | 0.511 | 0.429 | 0.610 | 0.638 |
| 2 | TOSV_Tunisie2010_T166_Partial | 0.000 | | 0.028 | 0.051 | 0.134 | 0.119 | 0.119 | 0.124 | 0.217 | 0.236 | 0.229 | 0.221 | 0.217 | 0.195 | 0.206 | 0.193 | 0.198 | 0.197 | 0.226 | 0.200 | 0.202 | 0.462 | 0.462 | 0.433 | 0.454 | 0.431 | 0.545 | 0.641 |
| 3 | TOSV_ISSPhl3_NC006318 | 0.052 | 0.029 | | 0.026 | 0.131 | 0.141 | 0.144 | 0.130 | 0.238 | 0.236 | 0.229 | 0.215 | 0.236 | 0.376 | 0.218 | 0.209 | 0.216 | 0.212 | 0.228 | 0.240 | 0.223 | 0.521 | 0.519 | 0.560 | 0.515 | 0.446 | 0.611 | 0.649 |
| 4 | TOSV_France_Marseille_DQ462405 | 0.038 | 0.038 | 0.025 | | 0.125 | 0.125 | 0.125 | 0.121 | 0.246 | 0.254 | 0.254 | 0.228 | 0.232 | 0.206 | 0.232 | 0.228 | 0.213 | 0.210 | 0.243 | 0.228 | 0.213 | 0.471 | 0.460 | 0.433 | 0.423 | 0.429 | 0.520 | 0.636 |
| 5 | TOSV_H/IMTSSA_FJ153286 | 0.168 | 0.168 | 0.149 | 0.114 | | 0.008 | 0.022 | 0.005 | 0.245 | 0.245 | 0.241 | 0.204 | 0.198 | 0.207 | 0.234 | 0.228 | 0.204 | 0.201 | 0.240 | 0.226 | 0.204 | 0.463 | 0.466 | 0.424 | 0.444 | 0.452 | 0.540 | 0.627 |
| 6 | TOSV_France_AR_EF656361 | 0.226 | 0.180 | 0.242 | 0.114 | 0.000 | | 0.036 | 0.012 | 0.246 | 0.248 | 0.241 | 0.199 | 0.228 | 0.380 | 0.218 | 0.217 | 0.205 | 0.212 | 0.246 | 0.259 | 0.214 | 0.522 | 0.524 | 0.567 | 0.510 | 0.457 | 0.609 | 0.652 |
| 7 | TOSV_Spain_EsPHGR40_EF120631 | 0.229 | 0.165 | 0.247 | 0.101 | 0.020 | 0.071 | | 0.017 | 0.240 | 0.245 | 0.238 | 0.185 | 0.216 | 0.382 | 0.226 | 0.217 | 0.208 | 0.212 | 0.233 | 0.258 | 0.216 | 0.531 | 0.529 | 0.571 | 0.518 | 0.452 | 0.608 | 0.649 |
| 8 | TOSV_Spain_AY705933 | 0.134 | 0.167 | 0.113 | 0.101 | 0.010 | 0.007 | 0.014 | | 0.252 | 0.248 | 0.245 | 0.192 | 0.227 | 0.217 | 0.234 | 0.229 | 0.215 | 0.213 | 0.246 | 0.244 | 0.215 | 0.499 | 0.505 | 0.457 | 0.483 | 0.462 | 0.566 | 0.631 |
| 9 | Massilia_W_EU725773 | 0.327 | 0.309 | 0.332 | 0.266 | 0.267 | 0.327 | 0.321 | 0.310 | | 0.058 | 0.046 | 0.199 | 0.199 | 0.251 | 0.238 | 0.243 | 0.227 | 0.247 | 0.210 | 0.258 | 0.234 | 0.476 | 0.479 | 0.450 | 0.481 | 0.409 | 0.552 | 0.659 |
| 10 | Massilia_BM_DQ656077 | 0.258 | 0.250 | 0.250 | 0.278 | 0.207 | 0.215 | 0.207 | 0.217 | 0.076 | | 0.012 | 0.206 | 0.202 | 0.236 | 0.230 | 0.233 | 0.215 | 0.236 | 0.218 | 0.242 | 0.215 | 0.451 | 0.445 | 0.435 | 0.482 | 0.397 | 0.515 | 0.619 |
| 11 | Massilia_BP_DQ656078 | 0.239 | 0.242 | 0.242 | 0.278 | 0.198 | 0.196 | 0.198 | 0.209 | 0.066 | 0.011 | | 0.204 | 0.201 | 0.231 | 0.229 | 0.232 | 0.214 | 0.235 | 0.210 | 0.238 | 0.214 | 0.451 | 0.444 | 0.426 | 0.481 | 0.394 | 0.508 | 0.615 |
| 12 | PNQV_P6B2_2008_GQ155520 | 0.227 | 0.241 | 0.218 | 0.190 | 0.238 | 0.227 | 0.244 | 0.235 | 0.261 | 0.217 | 0.220 | | 0.018 | 0.227 | 0.253 | 0.244 | 0.192 | 0.226 | 0.228 | 0.217 | 0.192 | 0.465 | 0.471 | 0.450 | 0.465 | 0.425 | 0.515 | 0.605 |
| 13 | PNQV_P1B4_2008_FJ848987 | 0.332 | 0.317 | 0.337 | 0.203 | 0.218 | 0.316 | 0.321 | 0.268 | 0.286 | 0.207 | 0.209 | 0.017 | | 0.251 | 0.268 | 0.255 | 0.210 | 0.248 | 0.231 | 0.246 | 0.221 | 0.479 | 0.484 | 0.472 | 0.472 | 0.425 | 0.556 | 0.636 |
| 14 | SFNV_Poona_HM566178 | 0.581 | 0.295 | 0.569 | 0.215 | 0.198 | 0.560 | 0.566 | 0.239 | 0.337 | 0.247 | 0.239 | 0.244 | 0.316 | | 0.155 | 0.172 | 0.166 | 0.003 | 0.215 | 0.258 | 0.169 | 0.556 | 0.551 | 0.491 | 0.562 | 0.429 | 0.635 | 0.654 |
| 15 | Cyprus_R3_EF201832 | 0.301 | 0.295 | 0.316 | 0.266 | 0.277 | 0.316 | 0.316 | 0.261 | 0.357 | 0.250 | 0.253 | 0.269 | 0.383 | 0.240 | | 0.072 | 0.133 | 0.155 | 0.200 | 0.247 | 0.136 | 0.458 | 0.464 | 0.435 | 0.489 | 0.437 | 0.564 | 0.659 |
| 16 | SFNV_Namru_840055_EF201828 | 0.291 | 0.281 | 0.306 | 0.253 | 0.277 | 0.332 | 0.311 | 0.261 | 0.367 | 0.239 | 0.242 | 0.244 | 0.327 | 0.255 | 0.117 | | 0.129 | 0.172 | 0.205 | 0.240 | 0.120 | 0.458 | 0.468 | 0.439 | 0.481 | 0.437 | 0.568 | 0.652 |
| 17 | SFNV_Sabin_Italy_EF201829 | 0.316 | 0.302 | 0.311 | 0.253 | 0.228 | 0.286 | 0.286 | 0.239 | 0.347 | 0.207 | 0.209 | 0.185 | 0.286 | 0.219 | 0.173 | 0.163 | | 0.165 | 0.197 | 0.238 | 0.000 | 0.482 | 0.483 | 0.456 | 0.483 | 0.412 | 0.561 | 0.645 |
| 18 | SFNV_India_P-7101795_EF201830 | 0.327 | 0.295 | 0.306 | 0.215 | 0.198 | 0.286 | 0.296 | 0.239 | 0.337 | 0.239 | 0.242 | 0.244 | 0.316 | 0.000 | 0.240 | 0.255 | 0.219 | | 0.218 | 0.253 | 0.169 | 0.478 | 0.470 | 0.432 | 0.490 | 0.434 | 0.568 | 0.627 |
| 19 | TEHV_I47_GQ165523 | 0.266 | 0.269 | 0.269 | 0.253 | 0.257 | 0.275 | 0.259 | 0.269 | 0.259 | 0.247 | 0.228 | 0.333 | 0.343 | 0.257 | 0.222 | 0.250 | 0.269 | 0.259 | | 0.203 | 0.197 | 0.463 | 0.476 | 0.425 | 0.486 | 0.434 | 0.528 | 0.631 |
| 20 | SFNV_Yugoslavia_YU8-76_EF2018 | 0.327 | 0.302 | 0.327 | 0.253 | 0.267 | 0.357 | 0.347 | 0.289 | 0.403 | 0.250 | 0.253 | 0.303 | 0.383 | 0.352 | 0.347 | 0.327 | 0.337 | 0.352 | 0.269 | | 0.231 | 0.485 | 0.481 | 0.456 | 0.480 | 0.449 | 0.587 | 0.636 |
| 21 | SFNV_Sabin_Italy_AY705944 | 0.246 | 0.246 | 0.239 | 0.253 | 0.228 | 0.232 | 0.225 | 0.239 | 0.310 | 0.207 | 0.209 | 0.185 | 0.218 | 0.169 | 0.113 | 0.099 | 0.000 | 0.169 | 0.269 | 0.275 | | 0.476 | 0.485 | 0.459 | 0.499 | 0.412 | 0.542 | 0.637 |
| 22 | SFSV_Sabin_EF201822 | 0.746 | 0.606 | 0.756 | 0.570 | 0.564 | 0.751 | 0.752 | 0.634 | 0.686 | 0.581 | 0.587 | 0.597 | 0.644 | 0.765 | 0.654 | 0.634 | 0.665 | 0.660 | 0.587 | 0.654 | 0.627 | | 0.073 | 0.551 | 0.325 | 0.466 | 0.580 | 0.654 |
| 23 | Cyprus_virus_GU119908 | 0.747 | 0.606 | 0.748 | 0.570 | 0.554 | 0.747 | 0.743 | 0.627 | 0.686 | 0.581 | 0.587 | 0.588 | 0.654 | 0.761 | 0.670 | 0.649 | 0.660 | 0.649 | 0.606 | 0.665 | 0.620 | 0.126 | | 0.568 | 0.332 | 0.460 | 0.580 | 0.644 |
| 24 | RVFV_NC014395 | 0.741 | 0.567 | 0.737 | 0.500 | 0.490 | 0.745 | 0.746 | 0.572 | 0.619 | 0.506 | 0.500 | 0.543 | 0.624 | 0.714 | 0.624 | 0.619 | 0.630 | 0.614 | 0.505 | 0.582 | 0.587 | 0.713 | 0.735 | | 0.548 | 0.461 | 0.627 | 0.702 |
| 25 | CFUV_PaAr814_EF201821 | 0.732 | 0.613 | 0.733 | 0.557 | 0.545 | 0.722 | 0.719 | 0.620 | 0.681 | 0.602 | 0.598 | 0.597 | 0.618 | 0.744 | 0.681 | 0.660 | 0.665 | 0.649 | 0.606 | 0.670 | 0.641 | 0.446 | 0.439 | 0.719 | | 0.402 | 0.576 | 0.662 |
| 26 | ARBV_Ph1_35M6_GQ165524 | 0.618 | 0.625 | 0.614 | 0.600 | 0.624 | 0.618 | 0.636 | 0.636 | 0.614 | 0.564 | 0.551 | 0.602 | 0.602 | 0.596 | 0.602 | 0.614 | 0.568 | 0.602 | 0.629 | 0.636 | 0.568 | 0.607 | 0.607 | 0.581 | 0.551 | | 0.471 | 0.676 |
| 27 | UUKV_M33551 | 0.809 | 0.748 | 0.809 | 0.714 | 0.701 | 0.813 | 0.813 | 0.732 | 0.741 | 0.670 | 0.667 | 0.670 | 0.730 | 0.823 | 0.767 | 0.751 | 0.762 | 0.741 | 0.724 | 0.767 | 0.732 | 0.764 | 0.775 | 0.792 | 0.767 | 0.581 | | 0.679 |
| 28 | Gouleako_virus_A5CI_2004_HQ541 | 0.897 | 0.922 | 0.900 | 0.892 | 0.870 | 0.889 | 0.897 | 0.879 | 0.907 | 0.884 | 0.894 | 0.899 | 0.907 | 0.879 | 0.901 | 0.907 | 0.914 | 0.901 | 0.910 | 0.901 | 0.902 | 0.865 | 0.856 | 0.925 | 0.885 | 0.915 | 0.921 | |
| | | *Amino acid sequences* | | | | | | | | | | | | | | | | | | | | | | | | | | | |

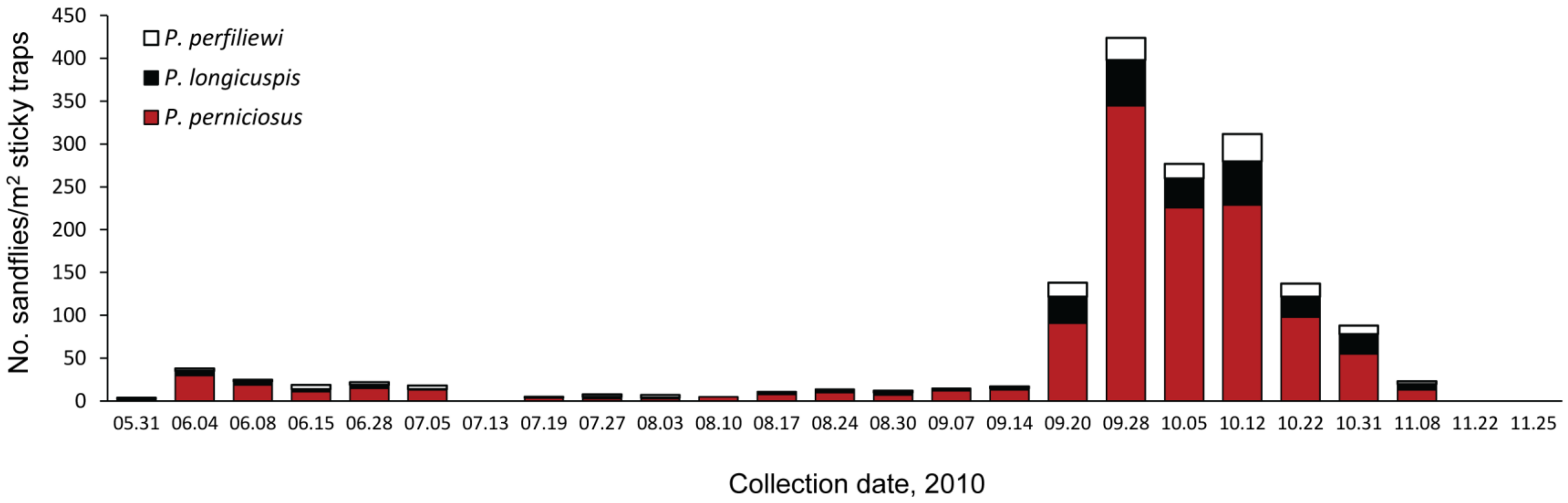


Technical Appendix Figure. Phenology of *Phlebotomus* spp. sandflies collected in Utique, Governorate of Bizerte, Tunisia, 2010.
